# Protein convertase subtilisin/Kexin type 9 inhibits hepatocellular carcinoma growth by interacting with GSTP1 and suppressing the JNK signaling pathway

**DOI:** 10.20892/j.issn.2095-3941.2020.0313

**Published:** 2022-01-15

**Authors:** Mingyan He, Jing Hu, Tingting Fang, Wenqing Tang, Bei Lv, Biwei Yang, Jinglin Xia

**Affiliations:** 1 Department of Gastroenterology, the First Affiliated Hospital of Nanchang University, Nanchang 330006, China; 2 Liver Cancer Institute, Zhongshan Hospital, Fudan University, Shanghai 200032, China; 3 Department of Cardiology, Jiangxi Provincial People’s Hospital Affiliated to Nanchang University, Nanchang 330006, China; 4 Department of Oncology, The First Affiliated Hospital of Wenzhou Medical University, Wenzhou 325000, China

**Keywords:** Hepatocellular carcinoma, growth, PCSK9, GSTP1, JNK

## Abstract

**Objective:**

Protein convertase subtilisin/Kexin type 9 (PCSK9) has been found to be closely associated with the occurrence and development of numerous tumors. However, the precise role of PCSK9 and its relationship to the development of hepatocellular carcinoma (HCC) remain largely unknown. This study aimed to clarify these issues.

**Methods:**

The expression levels of PCSK9 in HCC tissues and HCC cell lines were determined by the quantitative reverse transcription polymerase chain reaction, Western blot, and immunohistochemical analyses, and the effects of PCSK9 expression on HCC cell biological traits were investigated by overexpressing and downregulating PCSK9 expression *in vivo* and *in vitro*. Additionally, the mechanism by which PCSK9 mediated dissociation of glutathione S-transferase Pi 1 (GSTP1) dimers and phosphorylation of the Jun N-terminal kinase (JNK) pathway components were investigated.

**Results:**

PCSK9 expression levels were significantly lower in HCC tissues than in adjacent non-tumor samples. *In vivo* and *in vitro* experiments suggested that PCSK9 inhibited HCC cell proliferation and metastasis. Further analysis showed that PCSK9 interacted with GSTP1 and promoted GSTP1 dimer dissociation and JNK signaling pathway inactivation in HCC cells. Moreover, the relationships between PCSK9 protein expressions and clinical outcomes were investigated. The PCSK9-lo group displayed a significantly shorter overall survival (OS; median OS: 64.2 months *vs.* 83.2 months; log-rank statistic: 4.237; *P* = 0.04) and recurrence-free survival (RFS; median RFS: 26.5 months *vs.* 46.6 months; log-rank statistic: 10.498; *P* = 0.001) time than the PCSK9-hi group.

**Conclusions:**

PCSK9 inhibited HCC cell proliferation, cell cycle progression, and apoptosis by interacting with GSTP1 and suppressing JNK signaling, suggesting that PCSK9 might act as a tumor suppressor and be a therapeutic target in HCC patients.

## Introduction

Hepatocellular carcinoma (HCC) is one of the most common malignant tumors and is characterized by insidious onset, rapid progression, and high mortality. Recently, the management of hepatitis virus carriers and therapeutic advances, including curative hepatectomy, liver transplantation, local treatment, radiation, and molecular targeted therapy, have provided great benefits to patients with HCC. However, the 5-year survivals of small HCC and advanced HCC are still only approximately 60% and 10%, respectively. High aggressiveness, metastasis, and recurrence are key reasons for the poor prognoses. Therefore, it is critical to elucidate the molecular mechanisms underlying HCC progression and develop novel treatment strategies.

Protein convertase subtilisin/Kexin type 9 (PCSK9) is a member of the family of preprotein invertases, which encode secretory serine proteases composed of a signal peptide, predomain, catalytic domain, and C-terminal cysteine-histidine-rich domain. Its function is to regulate the posttranscriptional degradation of the low-density lipoprotein receptor (LDLR) by binding to it and promoting its translocation to acidic endosomes or lysosomes. PCSK9-mediated LDLR degradation leads to a decrease in the cholesterol uptake ability of cells and an increase in LDL cholesterol (LDL-C) levels and hypercholesterolemia. In addition, PCSK9 has been reported to play an important role in numerous biological processes, including the inflammatory response, the immune response, metabolism, the cell cycle, and apoptosis^1–3^. A previous study showed that PCSK9 expression was lower in HCC tissues than in paracancerous tissues^4^. Additionally, our previous study showed that Chinese kiwifruit root extract inhibited the development of hepatocarcinoma cells and that PCSK9 is the key molecule of this Chinese herbal extract responsible for suppressing liver cancer cell proliferation^5^. PCSK9 has also been found to be closely related to the occurrence and development of a variety of tumors, including melanoma, prostate cancer, and lung cancer^6–8^. However, the precise role of PCSK9 and its relationship to HCC development is largely unknown.

In this study, we investigated the expressions of PCSK9 in HCC cell lines and tumor samples and identified the mechanism by which HCC growth was related to changes in PCSK9 expression *in vitro* and *in vivo*. Using tissue microarrays (TMAs) containing HCC samples, we also identified the relationships between PCSK9 expression and clinicopathological parameters to evaluate its prognostic significance.

## Materials and methods

### Tissue specimens and TMA preparation

The mRNA and protein samples from human HCC tissues (*n* = 48) and matched adjacent nontumor samples (*n* = 48) were collected from patients who had undergone curative hepatectomy at Zhongshan Hospital affiliated to Fudan University.

TMA preparation was performed as described previously^9^. Primary HCC samples for the TMAs were obtained postoperatively from 241 patients with HCC whose 10-year follow-up data were available at Zhongshan Hospital affiliated to Fudan University. The study was approved by the Zhongshan Hospital Research Ethics Committee and was conducted in accordance with the Declaration of Helsinki (Approval No. B2020-277R). Informed consent was obtained from each patient. Follow-up procedures were previously described^9^.

### Immunohistochemistry (IHC) assays

In a typical procedure, after rehydration and antigen retrieval, cell slides were probed with primary antibodies against PCSK9 (1:100; Abcam, Cambridge, UK) at 4 °C overnight and were then incubated with horseradish peroxidase-conjugated IgG (1:500; Invitrogen, Carlsbad, CA, USA) at 37 °C for 30 min. Finally, the slides were stained with 3,3′-diaminobenzidine and counterstained with Mayer’s hematoxylin. All IHC staining was independently assessed by 2 experienced pathologists who were blinded to the patients’ outcomes. Five high power fields (200× magnification) were randomly selected. Based on the IHC staining percentage and intensity of positive cells counted in each core, immunoreactivity was graded on a scale from 0 to 3 points (0, no staining; 1, weak staining; 2, strong staining; and 3, very strong staining). All HCC samples were stratified based on the IHC staining scores into the low-PCSK9 group (PCSK9-lo; scores of 0–1) or the high-PCSK9 group (PCSK9-hi; scores of 2–3)^10^.

### Cell culture

The human HCC cell lines, HepG2 and Huh7, were purchased from the Cell Bank of the Institute of Biochemistry and Cell Biology, Chinese Academy of Sciences (Shanghai, China). The HCC cell lines, MHCC-97H (97H) and MHCC-97L (97L), were established at the Liver Cancer Institute^11^. Cells were cultured in Dulbecco’s Modified Eagle’s Medium supplemented with 10% (v/v) fetal bovine serum (FBS; Gibco, Gaithersburg, MD, USA), 10 µg/mL streptomycin sulfate, and 100 µg/mL penicillin G at 37 °C in a humidified atmosphere containing 5% CO_2_.

### Total RNA isolation and quantitative reverse transcription polymerase chain reaction (qRT-PCR)

Total RNA was isolated from HCC cells and HCC tissues using TRIzol reagent (Invitrogen) according to the manufacturer’s protocol. Complementary DNA was synthesized from 500 ng of RNA using a Reverse Transcription Reagent Kit (TaKaRa, Shiga, Japan). Real-time qRT-PCR analysis was performed using an ABI7500 real-time fluorescence measurement system (Applied Biosystems, Foster City, CA, USA) and a PCR amplification kit (TaKaRa). The sequences of the primers were as follows: PCSK9 forward, 5′-GAGAGCTGCGTTGCGTTTGTTTAC-3′ and reverse, 5′-CCGTTCTTCAGGGAGGCTACCACT-3′; β-actin forward, 5′-TTGCCGACAGGATGCAGAAG-3′ and reverse, 5′-CAGCGAGGCCAGGATGGAGC-3′. Expression values were derived from at least 3 independent experiments performed in duplicate and normalized to those of β-actin. The 2^−ΔΔCt^ method was used for data analysis^12^.

### Western blot

Procedures were performed as described previously^13^. All experiments were performed in triplicate. A nonreducing polyacrylamide gel electrophoresis (PAGE) gel and protein samples were prepared as follows. The nonreducing gel was prepared according to the second part of the concentrated resolving and separating gel preparation scheme without the addition of SDS. After total protein was extracted, it was diluted 4:1 with 5× nondenaturing and nonreducing buffer without boiling and was directly loaded onto the nonreducing PAGE gel. Antibodies against PCSK9 and glutathione S-transferase Pi 1 (GSTP1; Abcam) were used for Western blot.

### Cell viability assay

HCC cell viability was assessed using Cell Counting Kit-8 (CCK-8) assay kits (Dojindo, Nagasaki, Japan) as described previously^5^. In brief, 4,000 cells were seeded into 96-well culture plates and allowed to attach for 6 h. Then, after 0, 24, 48, and 72 h, 100 mL of CCK-8 assay buffer was added to each well. The optical density (450 nm) was measured in each well using a microplate reader (Bio Tek, Winooski, VT, USA) according to the manufacturer’s instructions. Three independent experiments were performed.

### Cell cycle analysis and apoptosis assay

As previously described^14^, cultured cells (1 × 10^6^) were collected and washed with phosphate-buffered saline (PBS). Cell pellets were fixed with 70% cold ethanol overnight at –20 °C. The fixed cells were washed in PBS and resuspended in Cell Cycle Reagent (Millipore, Burlington, MA, USA) at 5 × 10^5^ cells/mL and incubated in the dark for 15 min at room temperature. The cell suspensions were analyzed by flow cytometry to determine cell populations in different cell cycle phases.

Early apoptotic changes were detected by fluorescein isothiocyanate (FITC)-annexin V staining. Propidium iodide (PI) was used to discriminate apoptotic and necrotic cells among the annexin V-positive cells. HCC cells (1 × 10^6^) were washed and then resuspended in 100 µL of binding buffer solution (Annexin V-FITC Kit; Immunotech, Ocala, FL, USA). Annexin V-FITC (5 µL) and PI (5 µL) were then added to the cell suspension for a 10 min incubation prior to fluorescence-activated cell sorting (FACS) analysis. FACS files were analyzed using FlowJo, version 9.5.2 (Tree Star, Ashland, OR, USA).

### Cell migration and Matrigel invasion assays

Cell migration and invasion assays were performed using 24-well Transwell plates (8 µm pore size; Millipore) with or without a Matrigel coating (BD Biosciences, San Jose, CA, USA). A total of 1 × 10^5^ cells were suspended in 100 µL of DMEM containing 1% FBS and seeded into the upper chamber, and 600 µL of DMEM containing 10% FBS was placed in the lower chamber. After 48 h of incubation, the Matrigel and cells remaining in the upper chamber were removed using cotton swabs. Cells on the lower surface of the membrane were fixed with 4% paraformaldehyde and stained with Giemsa. Cells in 5 microscopic fields were counted and photographed (200× magnification). All experiments were performed in triplicate.

### Lentivirus construction and transfection

The PCSK9-shRNA, control shRNA, PCSK9 overexpression, and control overexpression lentiviral constructs were purchased from Shanghai GeneChem, (Shanghai, China). The lentiviral vectors, Hu6-MCS-Ubi-EGFP-IRES-puromycin and Ubi-MCS-3FLAG-CMV-EGFP, were used to generate recombinant lentiviruses. PCSK9-targeting shRNAs were designed as follows: shRNA-1, 5′-GCATGTCTTCCATGGCCTTCT-3′; shRNA-2, 5′-GCTTCCTGGTGAAGATGAGTG-3′; shRNA-3, 5′-GGTCACCGACTTCGAGAATGT-3′; and nontargeting control shRNA sequence, 5′-TTCTCCGAACGTGTCACGT-3′. Stable PCSK9 knockdown and upregulation were confirmed by qRT-PCR and Western blot.

Plasmids expressing GSTP1-siRNA were purchased from GeneChem. The sequence of the small interfering RNA (siRNA) sequence targeting GSTP1 was as follows: 5′-ACACCGTGGTCTATTTCCCAGTTCG-3′. Cells were transfected with plasmids using Lipofectamine 2000 (Invitrogen) according to the manufacturer’s protocol.

### Co-immunoprecipitation (co-IP) and mass spectrometry (IP/MS)

The procedures were performed as described previously^10,15^. Cultured cells were lysed in RIPA buffer (20 mM Tris, pH 8.0), 137 mM NaCl, 1% NP40, 10% glycerol, and 2 mM EDTA). The lysates were then mixed with the anti-PCSK9 monoclonal antibody (mAb), anti-GSTP1 mAb, and anti-IgG (Abcam) and 20 µL of protein A-agarose beads (Santa Cruz Biotechnology, Santa Cruz, CA, USA) for overnight incubation. The protein-antibody complexes that were recovered on the beads were subjected to mass spectrometry analysis after separation by SDS-PAGE. Candidate proteins interacting with PCSK9 were validated by Western blot.

### Immunofluorescence staining

HCC cells grown on coverslips were fixed with 3% paraformaldehyde at 4 °C overnight. To block nonspecific antibody binding, the cells were incubated with 3% bovine serum albumin for 30 min at room temperature. The cells were then incubated with primary antibodies against PCSK9 (1:100, Abcam) and GSTP1 (1:100, Abcam) at 4°C overnight and probed with secondary antibodies at room temperature for another 2 h. The cells were then mounted with ProLong^®^ Gold Antifade Reagent with 4′,6-diamidino-2-phenylindole (Life Technologies, Carlsbad, CA, USA) and immediately observed using a fluorescence microscope.

### The nude mouse model and metastasis assay

HCC cells (1.2 × 10^8^ cells in 0.1 mL of PBS) were injected subcutaneously into the left dorsal flanks of 8-week-old male BALB/c nude mice. These mice were randomly divided into 4 groups before injection. After the tumors were visible, the tumor size was measured every week for 4 weeks. The nude mice were sacrificed, and the subcutaneous tumors were excised and cut into tumor tissue blocks with a size of approximately 1 mm × 1 mm × 1 mm as the tumor source for subsequent orthotopic liver implantation. The mice implanted with the tumor blocks were sacrificed after 8 weeks, and the number of metastatic tumors was assessed by double blind evaluation. The research was reviewed and approved by the Zhongshan Hospital Research Ethics Committee before it was performed (Approval No. 2016-109).

### Statistical analysis

Statistical analyses were performed using SPSS statistical software for Windows, version 19.0 (SPSS, Chicago, IL, USA). The Kaplan-Meier method was used for survival analysis, and differences were compared using the log-rank test. Pearson’s χ^2^ test or Fisher’s exact test were used to compare qualitative variables, and Student’s *t*-test was used for quantitative variables. A Cox proportional hazards model was used for multivariate analysis. The level of significance was set at *P* < 0.05 for all tests.

## Results

### PCSK9 is downregulated in human HCC tissues

**Figure 1:**
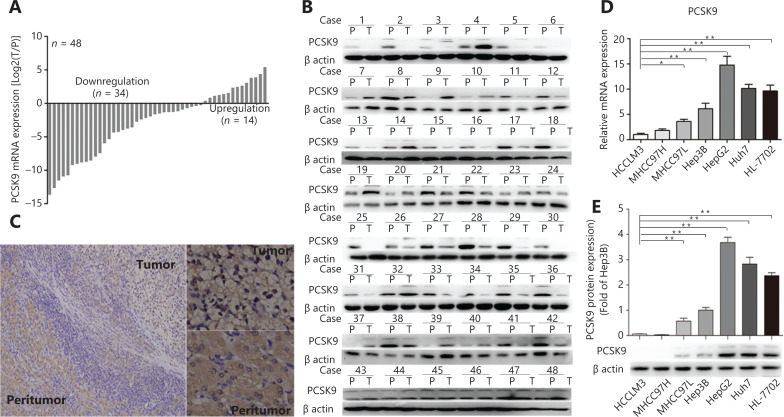
PCSK9 is downregulated in human hepatocellular carcinoma (HCC) tissues. (A, B, C) Relative PCSK9 mRNA and protein levels were significantly decreased in HCC tissues compared with adjacent non-tumor tissues (*n* = 48). (C) Representative images from tumor sample stained with PCSK9, Magnification, 100× (left), 400× (right). (D) Relative PCSK9 mRNA levels in different HCC cell lines and an immortalized normal liver cell line. (E) PCSK9 protein levels in different HCC cell lines and an immortalized normal liver cell line. The data are presented as the means ± SD of triplicate samples; *P < 0.05; **P < 0.01.

We first determined the levels of PCSK9 mRNA and protein in 48 human HCC tissues and adjacent non-tumor samples using qRT-PCR, Western blot analysis, and immunohistochemistry. PCSK9 expression was decreased in most HCC samples relative to non-tumor samples (*n* = 34 *vs. n* = 14; **Figure 1A–1C**). PCSK9 expression was further assessed in various HCC cell lines with different metastatic potentials [HCCLM3 (LM3), 97H, 97L, Hep3B, HepG2, and Huh7] and the HL-7702 immortalized normal liver cell line (**Figure 1D–1E**). The expression of PCSK9 was lower in the LM3 and 97H cell lines (relatively high malignancy) than in the HepG2 and Huh7 cell lines (relatively low malignancy) or in the immortalized hepatocyte line. These results suggested that PCSK9 was important during hepatocarcinogenesis and HCC development.

### PCSK9 affects the biological behavior of HCC cells

**Figure 2:**
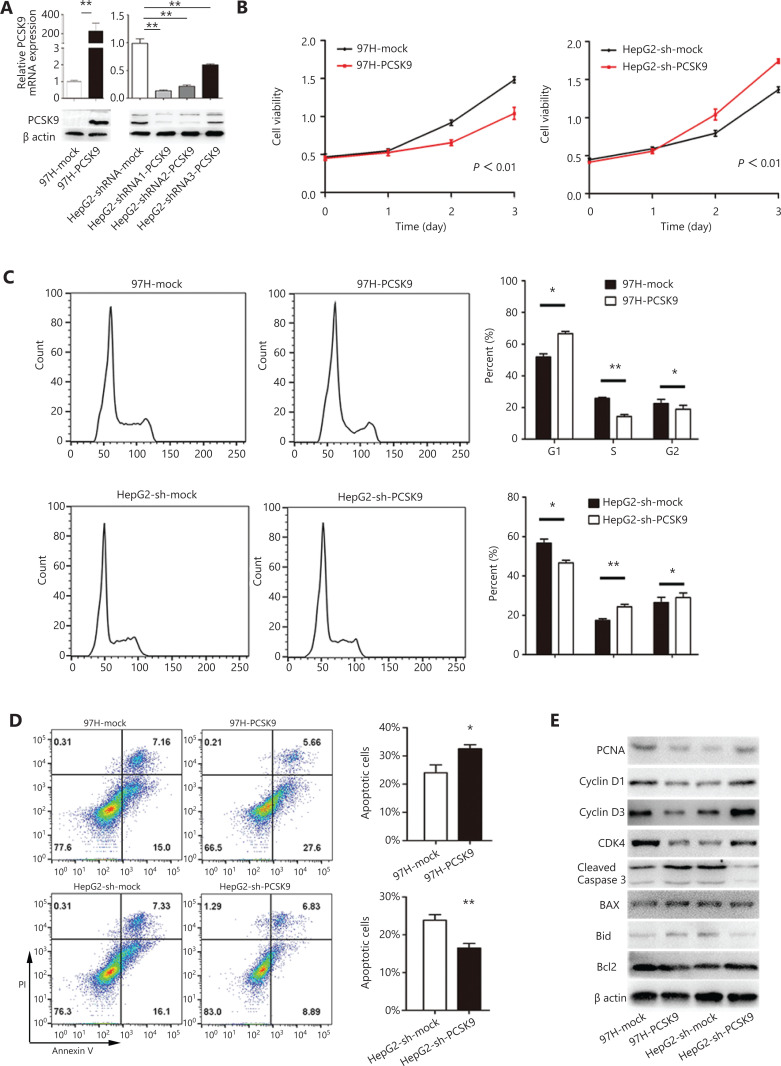
The effects of PCSK9 on cell proliferation, cell cycle phase distribution and apoptosis. (A) PCSK9 overexpression in 97H cells and successful knockdown of PCSK9 in HepG2 cells were confirmed by Western blot and qRT-PCR. (B) The proliferations of 97H and HepG2 cells were significantly changed after knockdown and overexpression of PCSK9, respectively. (C) Representative results of cell cycle analysis by flow cytometry after propidium iodide (PI) staining. (D) Representative results of apoptosis analysis by flow cytometry after dual staining with annexin V and PI. (E) Western blot analysis shows the levels of a proliferation marker (proliferating cell nuclear antigen), apoptosis markers (Bcl-2, Bax, Bid, and cleaved caspase-3) and cell cycle proteins (cyclin D1, cyclin D3, and CDK4). The data are expressed as the mean ± SD of 3 independent experiments, each performed in triplicate; *P < 0.05, **P < 0.01.

To identify the biological function of PCSK9 in HCC development, lentivirus-mediated overexpression and knockdown of PCSK9 in HCC cells were performed. Because the expression of PCSK9 was relatively low in 97H cells and relatively high in HepG2 cells, we stably overexpressed PCSK9 in 97H cells (97H-PCSK9) and successfully knocked down PCSK9 in HepG2 cells (HepG2-shRNA-PCSK9) (**Figure 2A**). The shRNA1-PCSK9 was the most effective shRNA (*P* < 0.01), and was used in subsequent experiments.

Proliferation of the transfected cells was investigated using a CCK-8 kit. High expression of PCSK9 in 97H cells was associated with a decreased growth rate compared to that of the corresponding control cells (*P* < 0.01), whereas HepG2 cells transfected with anti-PCSK9 shRNA grew faster than the corresponding control cells (*P* < 0.01, **Figure 2B**). We further investigated whether the altered growth rate was associated with the cell cycle and/or apoptosis. PI staining showed that the cell cycle was arrested in G1 phase, with 66.6% of 97H-PCSK9 cells in G0/G1 phase compared to 51.9% of control cells (*P* < 0.05, **Figure 2C**). In contrast, PCSK9 knockdown in HepG2 cells significantly promoted cell cycle progression, with 46.6% of HepG2-shRNA-PCSK9 cells and 56.6% of control cells in G0/G1 phase (*P* < 0.05, **Figure 2C**). Additionally, annexin V/PI assays showed a significant increase in apoptosis in 97H-PCSK9 cells compared to the corresponding control cells (*P* < 0.05), whereas HepG2-shRNA-PCSK9 cells displayed a lower apoptosis percentage than the corresponding control cells (*P* < 0.01, **Figure 2D**). We also detected the expression levels of proliferation markers, apoptosis markers, and cell cycle proteins. Western blot analyses showed that overexpression of PCSK9 significantly inhibited the protein levels of proliferating cell nuclear antigen (PCNA), cyclin D1, cyclin D3, and Bcl-2, and increased those of Bax, Bid, and cleaved caspase-3. PCSK9 knockdown resulted in the opposite effects (**Figure 2E**).

**Figure 3:**
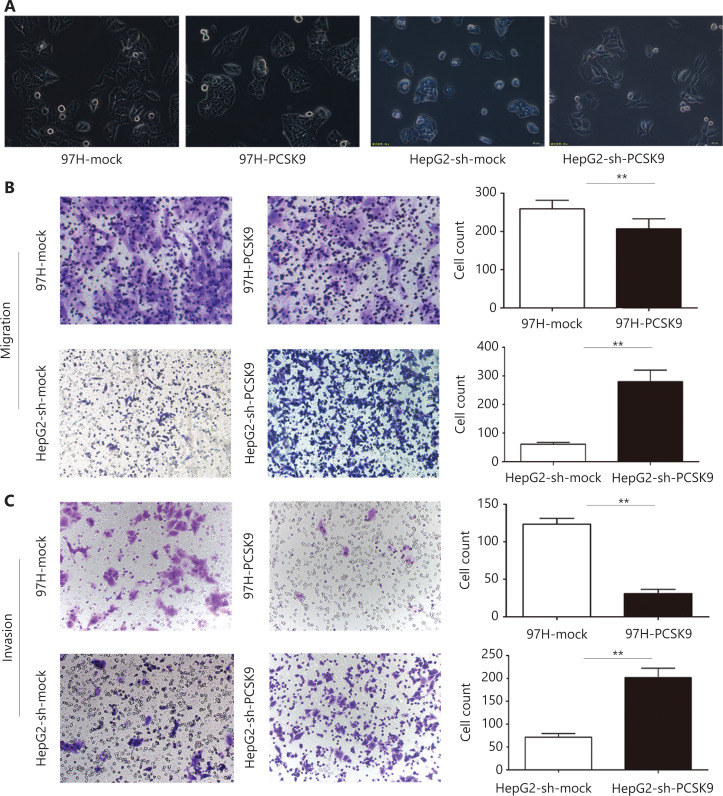
The effect of PCSK9 on the migration and invasion of hepatocellular carcinoma (HCC) cells. (A) Morphological analysis of HCC cells with knockdown and overexpression of PCSK9. (200×). (B) Migration and (C) invasive behavior were determined using Transwell Matrigel invasion assays after knockdown of PCSK9 in HCC cells. Data are expressed as the mean ± SD, with each experiment performed in triplicate; **P < 0.01. (200×).

Furthermore, we found that HepG2 cells transfected with anti-PCSK9 shRNA exhibited a more spindle-like morphology than the corresponding control cells, whereas high expression of PCSK9 in 97H cells was associated with a more epithelial-like morphology than the corresponding control cells (**Figure 3A**). *In vitro* migration and invasion assays showed that the numbers of migrated and invaded HepG2-sh-PCSK9 cells were significantly higher than those of the corresponding control cells (*P* < 0.01), whereas the numbers of migrated and invaded 97H-PCSK9 cells were significantly lower than those of 97H-mock cells (*P* < 0.01, **Figure 3B–3C**). These results indicated that PCSK9 inhibited HCC cell migration and invasion *in vitro*.

### Effects of PCSK9 on HCC tumor growth and metastasis *in vivo*

**Figure 4:**
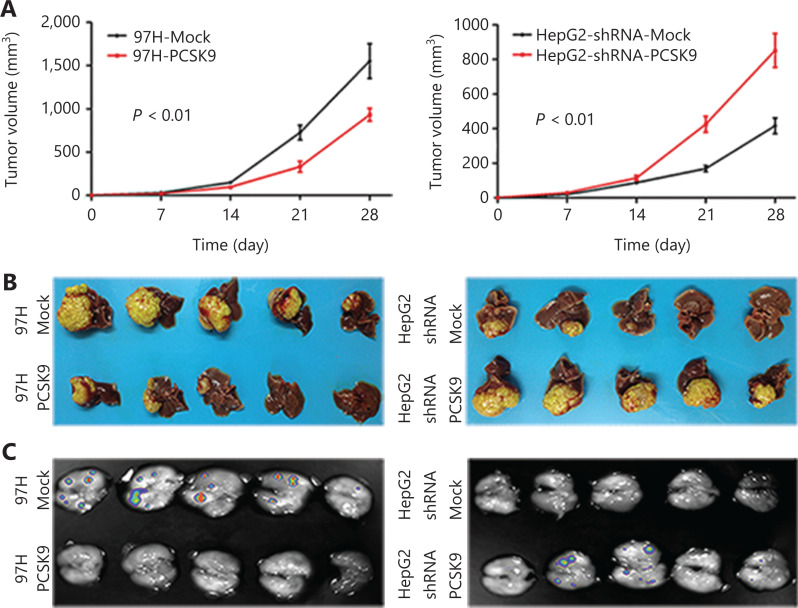
Effects of PCSK9 on hepatocellular carcinoma tumor growth and metastasis *in vivo*. (A) Average subcutaneous tumor volumes were plotted against time (days). (B) Macroscopic images of isolated liver tumors formed from 97H-GFP and HepG2-GFP cells. The results of statistical analyses using repeated measures analysis of variance are indicated. (C) Macroscopic images of lung tissue with metastatic foci of 97H-GFP and HepG2-GFP cells acquired using an *in vivo* imaging system.

The 97H cells stably expressing PCSK9 or control vector were injected subcutaneously into nude mice. Palpable tumors formed within 2 weeks. Tumor volumes were measured each week, and the mice were sacrificed 5 weeks after tumor cell injection. The average volume of the tumors formed from 97H cells stably transfected with PCSK9 was 0.902 ± 0.081 cm^3^, which was significantly smaller than those of tumors in the control group (1.572 ± 0.102 cm^3^; *P* < 0.01) (**Figure 4A**). The average volume of the tumors formed from HepG2 cells stably transfected with shRNA-PCSK9 was 0.815 ± 0.068 cm^3^, which was also significantly smaller than those of tumors in the control group (0.407 ± 0.032 cm^3^; *P* < 0.01) (**Figure 4A**).

To further determine the effects of PCSK9 on tumor growth and metastasis, we isolated the tumors described above to generate xenografts and implanted them into the livers of nude mice to establish orthotopic models. After 8 weeks, the average volume of 97H-PCSK9 orthotopic tumors was significantly smaller than that of tumors in the control group (**Figure 4B**), and the total number of lung metastatic lesions in the 97H-PCSK9 group was much lower than that in the control group (**Figure 4C**). These results suggested that PCSK9 acted as a tumor suppressor.

### PCSK9 interacts with GSTP1

**Figure 5:**
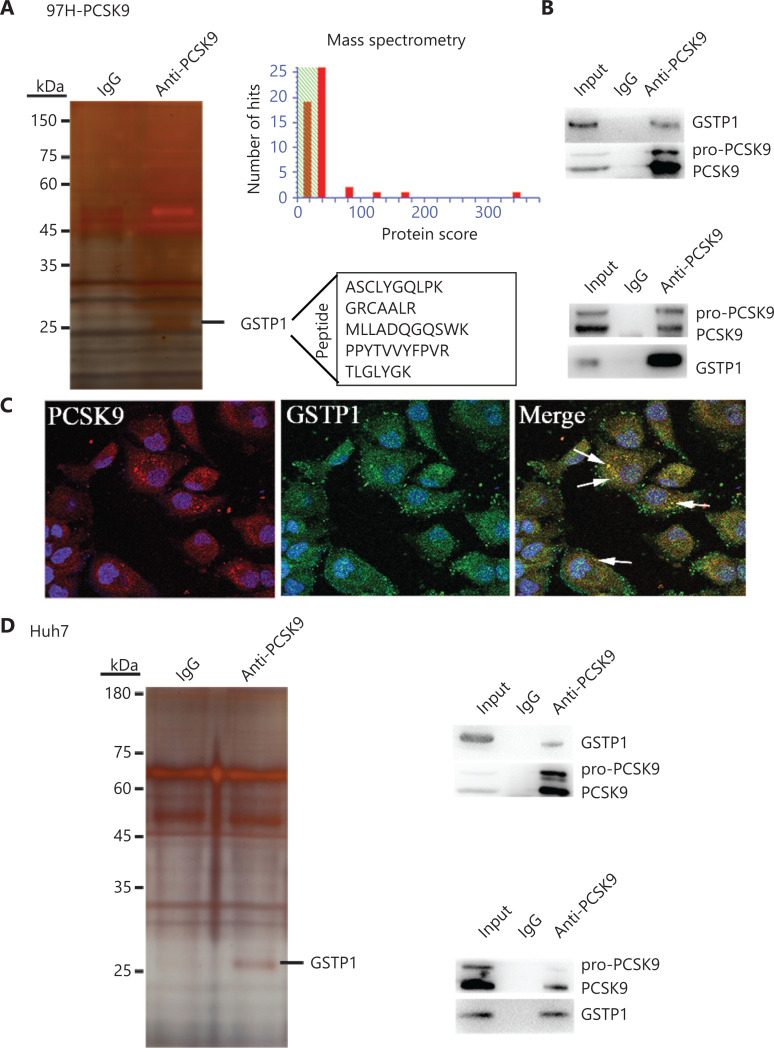
PCSK9 interacts with GSTP1. (A) Co-immunoprecipitation of PCSK9-binding proteins followed by mass spectrometry identified GSTP1 as a PCSK9-binding protein. (B) The presence of PCSK9 and GSTP1 in the immunoprecipitates was confirmed by Western blot with specific antibodies. (C) Immunofluorescence staining analysis showed that PCSK9 co-localized with GSTP1 in the cytoplasm. (D) The interaction between PCSK9 and GSTP1 was further verified in Huh7 cells.

To identify the mechanism by which PCSK9 inhibited HCC cell behavior, IP/MS was conducted to identify key factors associated with PCSK9 in 97H cells transfected with the PCSK9 vector and in Huh7 cells with naturally high levels of PCSK9. The PCSK9 protein complex was immunoprecipitated with an anti-PCSK9 mAb, and anti-IgG was used as a control to eliminate nonspecific binding. The co-immunoprecipitated proteins were visualized by silver staining after electrophoresis, and their amino acid sequences were identified by mass spectrometry. One factor identified was GSTP1 (**Figure 5A**). To validate the interaction between PCSK9 and GSTP1, co-immunoprecipitation using an anti-GSTP1 antibody was performed, and the Western blot results suggested that PCSK9 could in turn be co-immunoprecipitated with GSTP1 (**Figure 5B**). Similar results were obtained using Huh7 cells (**Figure 5D**). Moreover, co-expressed PCSK9 and GSTP1 were found to co-localize in 97H-PCSK9 cells (**Figure 5C**).

### PCSK9 promotes dissociation of GSTP1 dimers and inactivation of JNK signaling

**Figure 6:**
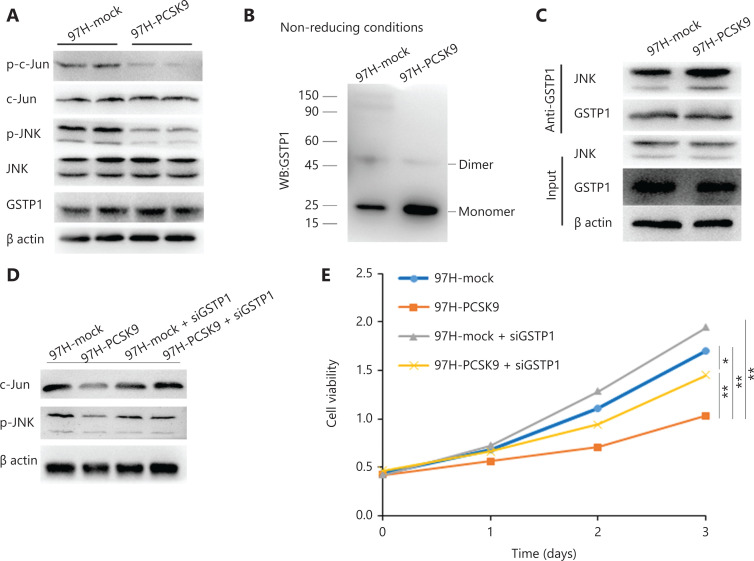
PCSK9 promotes dissociation of GSTP1 dimers and activity of the JNK signaling pathway. (A) GSTP1 expression and JNK signaling pathway activity were detected by Western blot. (B) The abundance of GSTP1 dimers in 97H cells was detected under nonreducing conditions. (C) The ability of GSTP1 to bind to JNK was evaluated by co-immunoprecipitation. (D) The effect of PCSK9 on JNK pathway activity was significantly inhibited by siGSTP1. (E) The effect of PCSK9 on HCC growth inhibition was decreased by siGSTP1. *P < 0.05; **P < 0.01.

GSTP1 plays an important role in the regulation of the Jun N-terminal kinase (JNK) signaling pathway and participates in cell proliferation, cell cycle progression, and apoptosis, among other processes^16,17^. To determine the role of GSTP1 in the tumor-suppressive effect of PCSK9, we detected GSTP1 protein expression and JNK signaling activity. No significant difference in GSTP1 expression was found, whereas the levels of phosphorylated JNK and phosphorylated c-Jun were reduced by overexpression of PCSK9 (**Figure 6A**). Previous studies have shown that monomeric GSTP1 exhibits higher JNK protein-binding activity than dimeric GSTP1, and can inhibit the phosphorylation of JNK and the activity of its downstream signaling pathway^18^. We found that the abundance of GSTP1 dimers in 97H-PCSK9 cells was significantly lower, but the abundance of GSTP1 monomers was significantly higher than that in 97H-mock cells, indicating that PCSK9 overexpression promoted dissociation of GSTP1 dimers (**Figure 6B**). We further examined the binding of GSTP1 to JNK and found that significantly more JNK protein was co-immunoprecipitated by the anti-GSTP1 antibody in 97H-PCSK9 cells than in 97H-mock cells (**Figure 6C**), suggesting that the PCSK9 protein promoted the transformation of GSTP1 dimers to GSTP1 monomers and enhanced the ability of GSTP1 to bind to the JNK protein. Moreover, the effects of PCSK9 on growth inhibition and JNK pathway activity were significantly enhanced in the presence of GSTP1, but decreased in the absence of GSTP1 induced by siGSTP1 (**Figure 6D–6E**). Taken together, our results suggested that PCSK9 inhibited HCC growth by interacting with GSTP1 and suppressing the JNK signaling pathway.

### Low PCSK9 protein expression in primary HCC tissues correlates with worse clinical outcomes

**Figure 7:**
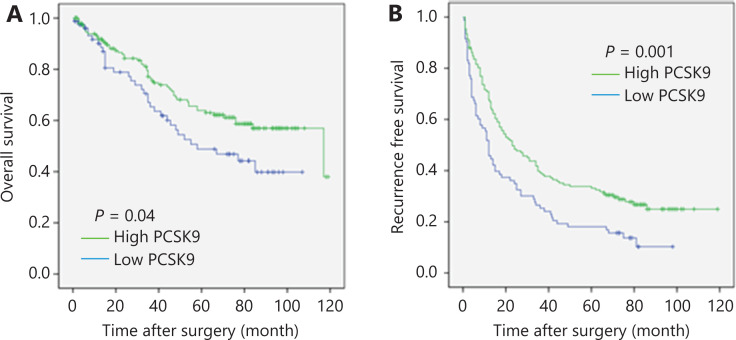
Correlation between PCSK9 protein expression and clinical outcomes. (A) Overall survival of patients with hepatocellular carcinoma (HCC) in the PCSK9-hi and PCSK9-lo groups. (B) Recurrence-free survival of patients with HCC in the PCSK9-hi and PCSK9-lo groups.

IHC staining of a TMA was performed to measure PCSK9 protein levels in HCC samples from 241 postoperative patients with HCC whose 10-year follow-up data were available. Six samples were damaged during array preparation, and the remaining 235 samples were analyzed. All HCC samples were stratified into PCSK9-hi or PCSK9-lo groups according to the IHC staining score. Comparisons of clinicopathological profiles between patients with HCC in the PCSK9-hi and PCSK9-lo groups are shown in **Supplementary Table S1**. Patients in the PCSK9-lo group displayed significantly shorter overall survival (OS) times (median OS: 64.2 months *vs* 83.2 months; log-rank statistic: 4.237; *P* = 0.04) and recurrence-free survival (RFS) times (median RFS: 26.5 months *vs* 46.6 months; log-rank statistic: 10.498; *P* = 0.001) than those in the PCSK9-hi group (**Figure 7**).

A high alpha-fetoprotein level, high carbohydrate antigen 19-9 (CA19-9) level, advanced Barcelona Clinic Liver Cancer (BCLC) stage, and lack of tumor encapsulation were found to be associated with worse OS and RFS using univariate analysis (**Table 1**). To further assess the correlation between the PCSK9 levels and other risk factors, a Cox proportional hazards analysis was performed. The results indicated that a low level of PCSK9 was an independent risk factor for worse OS [hazard ratio: 2.025; 95% confidence interval (CI): 1.384–2.961; *P* = 0.000] and decreased RFS time (hazard ratio: 1.749; 95% CI: 1.034–2.958; *P* = 0.037) (**Table 1**).

**Table 1:** Univariate and multivariate analyses of factors associated with overall survival and recurrence-free survival in hepatocellular carcinoma patients

Features	Overall survival				Recurrence-free survival			
	Univariate *P*	Multivariate			Univariate *P*	Multivariate		
		Hazard ratio	95% CI	*P*		Hazard ratio	95% CI	*P*
Age (≤ 54 *vs*. > 54 years)	0.202			NA	0.242			NA
Gender (female *vs*. male)	0.905			NA	0.514			NA
Hepatitis B history: yes *vs.* no	0.776			NA	0.321			NA
HbeAg (positive *vs*. negative)	0.159			NA	0.119			NA
Liver cirrhosis: yes *vs*. no	**0.039**	1.928	1.133–3.281	**0.015**	0.115			NA
AFP (< 400 *vs.* ≥ 400 ng/mL)	**0.000**			NS	**0.000**	0.486	0.275–0.861	**0.013**
Preoperative ALT (< 75 *vs.* ≥ 75 U/L)	**0.001**			NS	0.551			NA
BCLC stage (A *vs*. B/C/D)	**0.000**	0.490	0.335–0.717	**0.000**	**0.000**	0.559	0.327–0.958	**0.034**
Tumor encapsulation (yes *vs*. no)	**0.008**	1.718	1.166–2.531	**0.006**	**0.035**			NS
Microvascular invasion (yes *vs.* no)	0.171			NA	**0.008**			NS
Tumor differentiation (I/II *vs*. III/IV)	0.710			NA	0.523			NA
CA199 (< 37 *vs.* ≥ 37 U/mL)	**0.000**	0.560	0.335–1.297	**0.027**	**0.005**			NS
PCSK9 level (low *vs.* high)	**0.001**	2.025	1.384–2.961	**0.000**	**0.040**	1.749	1.034–2.958	**0.037**

## Discussion

**Figure 8:**
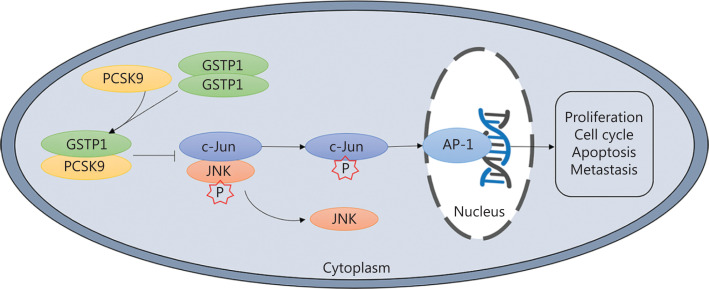
Representation of the experimental protocols.

In this study, we demonstrated that PCSK9 expression was lower in HCC tissues than in adjacent non-tumor samples. The results of *in vivo* and *in vitro* experiments suggested that PCSK9 inhibited HCC cell proliferation and lung metastasis. Further analysis showed that PCSK9 interacted with GSTP1 and promoted dissociation of GSTP1 dimers and inactivation of the JNK signaling pathway (**Figure 8**). Moreover, positive correlations between PCSK9 protein expression and clinical outcomes were confirmed. Together, these results suggested that PCSK9 acted as a tumor suppressor in HCC.

Previous studies have reported that the PCSK9 protein induces the degradation of many lipid-related protein receptors and other proteins, including LDLR, the very low-density lipoprotein receptor (VLDLR)^19^, apoE receptor 2^19^, CD36^20^, and CD81^21^. In addition, PCSK9 has been reported to be related to various biological pathways, such as those mediating the inflammatory response, the immune response, metabolism, the cell cycle, and apoptosis^1–3^, suggesting that PCSK9 is a multifunctional protein. However, only a few studies have reported a relationship between PCSK9 and cancer^6,8^. One study found a decrease in PCSK9 expression in human HCC tissues but did not clarify the specific significance of low PCSK9 expression levels in the initiation and development of HCC^4^. Our study provided the first demonstration that PCSK9 interacted with GSTP1, affected JNK phosphorylation levels, and inhibited HCC proliferation, which was highly important for further understanding the function of the PCSK9 protein and suggested that PCSK9 may be a therapeutic target for the treatment of HCC. However, the data from these mechanistic studies were not replicated in HepG2 cells. HepG2 is a hepatoblastoma cell line with relatively low GSTP1 protein expression, indicating that PCSK9 may affect the growth of HepG2 cells through other mechanisms, a possibility to be addressed in our future research. Additionally, whether the function of PCSK9, as an important gene regulating the intracellular transport and degradation of membrane LDLR, is affected by GSTP1 has not been reported. Further study of the effects of GSTP1 on the PCSK9/LDLR signaling pathway will contribute to our understanding of cellular cholesterol metabolism.

GSTP1 is an important subtype of human GST, which can catalyze the reduction of excessive levels of reactive oxygen species (ROS) and oxygen free radicals produced in organisms, to maintain the redox homeostasis in organisms. GSTP1 also regulates the mitogen-activated protein kinase (MAPK)-dependent cell cycle, proliferation, apoptosis, the inflammatory response, DNA damage repair, and other biological processes through its ligand-binding activity^16,17^. JNKs are members of the MAPK family. JNK serine/tyrosine kinase activity is activated under pressure or stimulation by ROS, radiation, inflammatory factors, and growth factors, among other stimulators, promoting the phosphorylation of its downstream substrates (such as c-Jun, c-Fos, and ATF2), as well as the formation of the activated protein-1 (AP-1) transcription complex^22,23^. In the tumor microenvironment, cancer cells undergo proto-oncogene transformation, and the JNK signaling pathway is usually activated and plays an important role in carcinogenesis and tumor progression^24–26^. *Via* its binding to the negatively charged C-terminal region of the JNK protein, GSTP1 is a natural inhibitor of the JNK signaling pathway^14,16,27^, and GSTP1 monomers have higher JNK protein-binding activity than GSTP1 dimers. When cells are stimulated by ROS, chemicals, and inflammatory factors, among other stimulators, GSTP1 is crosslinked to form dimers or multimers, relieving JNK inhibition and activating the JNK pathway^28^. Increased expression of GSTP1 has been detected in most tumor tissues and is closely related to tumorigenesis and development. In the present study, PCSK9 was found to interact with GSTP1 to affect the JNK signaling pathway activity and thus the biological function of HCC cells, consistent with the results of previous studies of GSTP1. However, the specific sites for binding among PCSK9, GSTP1, and JNK are unknown, and the role of JNK in PCSK9-mediated inhibition of HCC growth has not been verified, warranting further studies of the interaction domains and the role of JNK in PCSK9-mediated inhibition of HCC growth.

## Conclusions

The collective findings of our study provided the first evidence that PCSK9 acted as a tumor suppressor gene in HCC. The mechanistic link between PCSK9, GSTP1, and JNK signaling indicated that PCSK9 was a potential therapeutic target for suppressing HCC development and metastasis.
