# Supplementary material for: Protein convertase subtilisin/Kexin type 9 inhibits hepatocellular carcinoma growth by interacting with GSTP1 and suppressing the JNK signaling pathway
Source: Cancer Biol Med. 2022 Jan 15;19(1):90–103. doi: 10.20892/j.issn.2095-3941.2020.0313 (PMC8763006; doi:10.20892/j.issn.2095-3941.2020.0313)
Supplement: Supplementary file 1 [file cbm-19-090-s001.pdf]

Supplementary material

Table S1 Comparison of clinicopathological profiles between hepatocellular carcinoma patients with high PCSK9 levels and those with low PCSK9 levels

Variables	PCSK9 level		OR	95% CI	*P* Value
	High (*n* = 152)	Low (*n* = 83)			
	Number of patients	Number of patients			
Age (years)			1.002	0.555~1.808	0.996
≤ 54	65	36			
> 54	87	47			
Gender			1.212	0.524~2.802	0.654
Female	19	13			
Male	133	70			
Hepatitis B history			0.944	0.390~2.284	0.899
Present	130	74			
Absent	22	9			
HbeAg			0.906	0.490~1.673	0.751
Positive	44	28			
Negative	108	55			
Liver cirrhosis			0.454	0.244~0.843	0.012
Present	84	61			
Absent	68	22			
BCLC stage			1.318	0.734~2.367	0.355
A	90	42			
B/C/D	62	41			
Tumor encapsulation			1.184	0.627~2.236	0.602
None	85	45			
Complete	67	38			
Microvascular invasion			0.719	0.386~1.340	0.299
Positive	59	38			
Negative	93	45			
Tumor differentiation			1.100	0.595~2.036	0.761
I/II	96	50			
III/IV	56	33			
AFP level			1.350	0.677~2.692	0.394
< 400 ng/mL	115	54			
≥ 400 ng/mL	37	29			
CA199 level			1.169	0.525~2.606	0.702
< 37 U/mL	133	69			
≥ 37 U/mL	19	14			
